# Basic properties and information theory of Audic-Claverie statistic for analyzing cDNA arrays

**DOI:** 10.1186/1471-2105-10-310

**Published:** 2009-09-23

**Authors:** Peter Tiňo

**Affiliations:** 1 School of Computer Science, The University of Birmingham, Birmingham, B15 2TT, UK

## Abstract

**Background:**

The Audic-Claverie method [1] has been and still continues to be a popular approach for detection of differentially expressed genes in the SAGE framework. The method is based on the assumption that under the null hypothesis tag counts of the same gene in two libraries come from the same but unknown Poisson distribution. The problem is that each SAGE library represents only a single measurement. We ask: Given that the tag count samples from SAGE libraries are extremely limited, how useful actually is the Audic-Claverie methodology? We rigorously analyze the A-C statistic that forms a backbone of the methodology and represents our knowledge of the underlying tag generating process based on one observation.

**Results:**

We show that the A-C statistic and the underlying Poisson distribution of the tag counts share the same mode structure. Moreover, the K-L divergence from the true unknown Poisson distribution to the A-C statistic is minimized when the A-C statistic is conditioned on the mode of the Poisson distribution. Most importantly, the expectation of this K-L divergence never exceeds 1/2 bit.

**Conclusion:**

A rigorous underpinning of the Audic-Claverie methodology has been missing. Our results constitute a rigorous argument supporting the use of Audic-Claverie method even though the SAGE libraries represent very sparse samples.

## Background

It is of utmost importance for biologists to be able to analyze patterns of expression levels of selected genes in different tissues possibly obtained under different conditions or treatment regimes. Even subtle changes in gene expression levels can be indicators of biologically crucial processes such as cell differentiation and cell specialization [2]. Measurement of gene expression levels can be performed either via hybridization to microarrays, or by counting gene tags (signatures) using e.g. Serial Analysis of Gene Expression (SAGE) [3] or Massively Parallel Signature Sequencing (MPSS) [4] methodologies. The SAGE procedure results in a library of short sequence tags, each representing an expressed gene. The key assumption is that every mRNA copy in the tissue has the same chance of ending up as a tag in the library. Selecting a specific tag from the pool of transcripts can be approximately considered as sampling with replacement. The key step in many SAGE studies is identification of "interesting" genes, typically those that are differentially expressed under different conditions/treatments. This is done by comparing the number of specific tags found in the two SAGE libraries corresponding to different conditions or treatments. Several statistical tests have been suggested for identifying differentially expressed genes through comparing such digital expression profiles, e.g. [1,2,5,6].

Audic and Claverie [1] were among the first to systematically study the influence of random fluctuations and sampling size on the reliability of digital expression profile data. Typically, cDNA libraries contain a large number of different expressed genes and observing a given cDNA qualifies as a rare event [1]. For a transcript representing a small fraction of the library and a large number *N *of clones, the probability of observing *x *tags of the same gene will be well-approximated by the Poisson distribution parametrized by *λ *≥ 0.

(1)

The unknown parameter *λ *signifies the number of transcripts of the given type (tag) per *N *clones in the cDNA library. When comparing two libraries, it is assumed that under the null hypothesis of not differentially expressed genes the tag count *x *in one library comes from the same underlying Poisson distribution *P*(·|*λ*) as the tag count *y *in the other library. However, each SAGE library represents a single measurement only. From a purely statistical standpoint resolving this issue is potentially quite problematic. One can be excused for being rather skeptical about how much can actually be learned about the underlying unknown Poisson distribution from a single observation.

The key instrument of the Audic-Claverie approach is a distribution (*y|x*) over tag counts *y *in one library informed by the tag count *x *in the other library, under the null hypothesis that the tag counts are generated from the same but unknown Poisson distribution. (*y|x*) is obtained by Bayesian averaging (infinite mixture) of all possible Poisson distributions *P*(*y*|*λ'*) with mixing proportions equal to the posteriors *p*(*λ'*|*x*) under the flat prior over *λ*. When the two libraries are of the same size, we obtain [1]:

(2)

(3)

We will refer to (*y|x*) as *Audic-Claverie statistic *(A-C statistic) based on counts *x *and *y*. Note that (*y*|*x*) is symmetric, i.e. for *x, y *≥ 0, (*y|x*) = (*x|y*). Audic and Claverie [1] point out that this is a desirable property, since if the counts *x, y *are related to two libraries of the same size, they should be interchangeable when analyzing whether they come from the same underlying process or not. The A-C statistic (*y|x*) can be used e.g. for principled inferences, construction of confidence intervals, statistical testing etc. For further details regarding the derivation and mathematical treatment of the A-C statistic see [1].

Even though there have been further developments in comparison techniques for cDNA libraries (e.g. while Audic and Claverie [1] only deal with two libraries, Stekel et al. [7] suggest an approach to compare gene expressions across multiple cDNA libraries; for links to further approaches see [2]), the Audic-Claverie method has been and still continues to be a popular approach in current biological research, e.g. [8-17], with 427 citations (based on ISI Web of Knowledge), over 100 citations in the past 3 years. Given the widespread use of the Audic-Claverie method, it is somewhat surprising that a rigorous underpinning of the methodology has not yet been fully developed. Audic and Claverie did demonstrate the desirable behavior of their method through Monte Carlo simulations randomly sampling tags based on two experimentally obtained sequence tag distributions [1]. The rate of false alarm, e.g. how often random fluctuations in tag counts are interpreted as significant differences, was small for genes associated with small tag counts and increased for higher tag counts, but never exceeded the significance level of the test. Of course, one may argue that false alarm rate (false positives) is only one side of the story and ideally one would like to minimize both the false positive and false negative rates. The false negative rate quantifies how often significant differences get interpreted as just random fluctuations. However, of equal importance is the issue of why the Audic-Claverie approach seems to be well-behaved, e.g. when compared to an approach based on Ricker's confidence intervals (see [1]). In this contribution, we provide rigorous arguments as to why the Audic-Claverie method can be expected to work well, even though from the purely statistical standpoint one could be excused for being skeptical. We start by assuming that for a given gene there is a hidden (unobserved) underlying Poisson distribution generating the tag counts. We then go beyond simple Monte-Carlo-style verification by rigorously studying *how much *and *in what form *can be actually learned about the distribution in the Audic-Claverie framework, given a single observation provided by a SAGE library. In particular, we ask:

1. How natural is the A-C statistic's representation of the underlying unknown Poisson distribution governing the tag counts?

2. Given that the observed tag count sample is very limited, how well can the Audic-Claverie approach work, i.e. how well does the A-C statistic capture the underlying Poisson distribution?

## Methods

### Basic properties of the A-C statistic

In this section we answer the first question posed above. It turns out that the A-C statistic and the underlying Poisson distribution are quite similar in their nature: for any (integer) mean tag count *λ *≥ 1, the Poisson distribution *P*(·|*λ*) has two neighboring modes located at *λ *and *λ *- 1, with *P*(*λ*|*λ*) = *P*(*λ *- 1|*λ*). When it comes to the observed tag counts, given a count *x *≥ 1, the A-C statistic (*y|x*) has two neighboring modes, one located at *y *= *x*, the other at *y *= *x *- 1, with (*x|x*) = (*x *- 1|*x*). As in Poisson distribution, the values of (*y|x*) decrease as one moves away from the modes in both directions.

**Theorem 1 ***Let x, y and d be integers with ranges specified below. It holds:*

*1*. (*x|x*) > (*x *+ *d|x*) *for any x *≥ 0 *and d *≥ 1.

*2. For x *≥ 1, (*x|x*) = (*x *- 1|*x*).

*3*. (*x|x*) > (*x - d|x*) *for any x *≥ 2 *and *2 ≤ *d *≤ *x*.

Proof:

1. We have



In particular,



Hence,



Now, for *x *≥ 0, we have



This can be easily seen, as for *j *≥ 1, 2(*x *+ *j*) > 2*x *+ *j*. It follows that



2. and 3) For *d *≤ *x*,



Hence,

(4)

If *d *= 1,



When 2 ≤ *d *≤ *x*, we have for all *j *such that 1 ≤ *j *≤ *d *- 1,



This follows from 2(*x - d *+ *j*) *<*2*x - d *+ *j*, which can be easily verified, since for *j *∈ {1, 2,..., *d *- 1}, we have (*j - d*) > 2·(*j - d*).

For *j *= *d*, we have the equality (2*x - d *+ *j*)/(*x - d *+ *j*) = 2.

Finally, form (4),



Q.E.D

We have shown that after observing a count *x*, the A-C statistic expects counts *y *= *x *and *y *= *x *- 1 with the highest and equal probability. The other values of count *y *are, as one would naturally expect, less probable.

**Figure 1:**
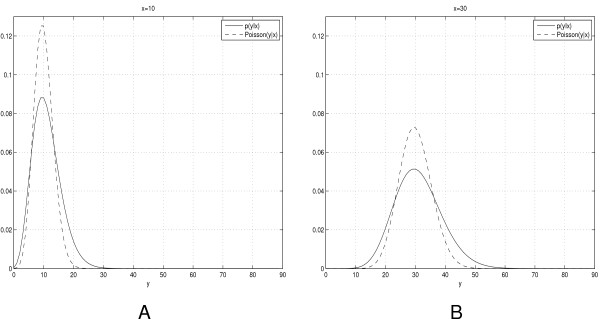
**A-C statistic vs. Poisson distribution**. Graphs of A-C statistic (*y|x*) (solid line) and the corresponding Poisson distribution *P*(*y|λ*) at *λ *= *x *(dashed line) for *x *= 10 (A) and *x *= 30 (B).

As an illustrative example we show in figure 1 plots of both the A-C statistic (*y|x*) and the corresponding Poisson distribution *P*(*y|λ*) at *λ *= *x *for two values of *x*, *x *= 10 and *x *= 30. As a result of Bayesian averaging in the A-C statistic, (*y|x*) is less peaked at its modes than the Poisson counterpart *P*(*y|x*). However, both (*y|x*) and *P*(*y|x*) have two modes located at *x *and *x *- 1.

### Information theory of the A-C statistic

We now answer, in the framework of information theory, the second question posed in the 'Background' section. Assume that there is some "true" underlying Poisson distribution *P*(*y|λ*) (1) over possible counts *y *≥ 0 with unknown parameter *λ*. In the same process, we first generate a count *x *and then use the A-C statistic (*y|x*) (3) to define a distribution over *y*, given the already observed count *x*. We ask: How different, in terms of Kullback-Leibler (K-L) divergence, are the two distributions over *y*? For the A-C statistic to work, one would naturally like (*y|x*) to be sufficiently representative of the true unknown distribution *P*(*y|λ*). In other words, one would expect *P*(*y|λ*) and (*y|x*) to be close, with the smallest "distance" at (*y|x *= *λ*) (for *λ *integer), that is, when count *x *is exactly equal to the expected tag count under the Poisson distribution *P*(*y|λ*). In this section we provide a quantitative answer to the above question and show, perhaps surprisingly, that the "statistical distance" between *P*(*y|λ*) and (*y|x*) is not minimized at *x *= *λ*, but it attains minimum at the mode of *P*(*y|λ*), i.e. when *x *= *λ *- 1.

First, define the K-L divergence from *P*(*y|λ*) to (*y|x*):

(5)

The divergence *D*(*λ*, *x*) has a nice information-theoretic interpretation: When the log is base 2, *D*(*λ*, *x*) expresses the number of bits of additional information one needs in order to fully specify (*y|x*), provided one has a perfect knowledge of *P*(*y|λ*). The divergence *D*(*λ*, *x*) is non-negative, with *D*(*λ*, *x*) = 0 if and only if the two distributions (*y|x*) and *P*(*y|λ*) coincide.

Naturally,



where



is the entropy of *P*(*y|λ*) and *E*_*Q*(*y*)_[*f *(*y*)] denotes the expectation of the quantity *f *(*y*) under the distribution *Q*(*y*).

We have



and so

(6)

where for each integer *d *≥ 0,

(7)

As discussed above, one would intuitively expect *D*(*λ*, *x*) to be minimal for *x *= *λ*, as then the conditioning count in the A-C statistic would be the mean of the underlying Poisson distribution. However, the mode of that Poisson distribution, *λ *- 1, is surrounded by enough probability mass to yield the following result:

**Theorem 2 ***For any integer λ *≥ 1, *it holds D*(*λ*, *λ*) > *D*(*λ*, *λ *- 1). *In other words*,



Proof: Using (6), we have

(8)

Now,



and by Jensen's inequality,



By (8), *D*(*λ*, *λ*) *- D*(*λ*, *λ *- 1) = log(2*λ*) + *F*(*λ*, *λ *- 1) *- F*(*λ*, *λ*), and since



we have *D*(*λ*, *λ*) *- D*(*λ*, *λ *- 1) > 0, implying *D*(*λ*, *λ*) *> D*(*λ*, *λ *- 1).

Q.E.D

We proceed our investigation by asking the following question: Given an underlying Poisson distribution *P*(*x|λ*), if we repeatedly generated a "representative" count *x *from *P*(*x|λ*), what would be the average divergence of the corresponding A-C statistic (*y|x*) from the truth *P*(*y|λ*)? In other words, we are interested in the quantity

(9)

**Lemma 3 ***For any λ *≥ 0,



Proof: Employing Malmstén's formula,

(10)

we write

(11)

The last equality follows from *E*_*P*(*y*|2λ)_[*y*] = 2*λ *and

(12)

Let us now evaluate



Using Malmstén's formula again, we obtain

(13)

Expansion similar to that in (12) leads to:

(14)

Plugging (14) into (13) we obtain (11).

Q.E.D

We will now show that up to terms of order *O*(*λ*^-1^), the expected divergence of A-C statistic (*y|x*)] from the true underlying Poisson distribution *P*(*y|λ*) is equal to (1/2) log 2.

**Theorem 4 ***Consider an underlying Poisson distribution P*(·|*λ*) *parametrized by some λ > *0. *Then*



Proof: Since

(15)

and

(16)

we have



By lemma 3,

(17)

We next approximate the terms *F*(*λ*, 0) and *F*(2λ, 0). To that end, note that the entropy *H*[*P*(*y|λ*)] can be approximated as [18]



Hence,

(18)

By the same token

(19)

Plugging (18) and (19) into (17) we obtain



Q.E.D

In fact, one can obtain a more precise characterization of the expected divergence *ε*(*λ*) by using a higher order entropy expansion (for log base 2):



After expressing *F*(*λ*, 0) and *F*(2λ, 0) in the style of (18) and (19), respectively, we obtain an expression for the expected divergence measured in bits:

(20)

**Figure 2:**
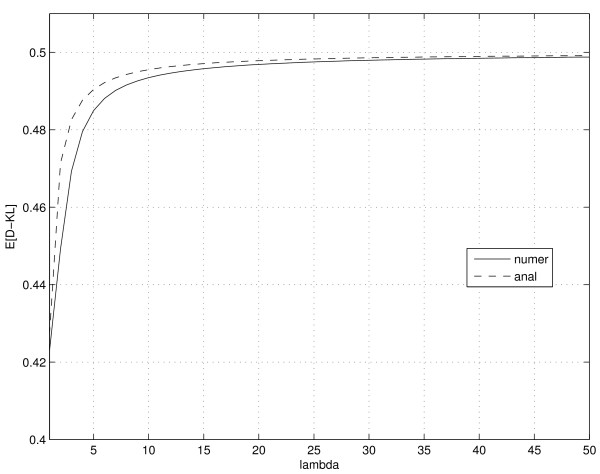
**Expected K-L divergence from the underlying Poisson distribution to A-C statistic**. Expected K-L divergence *ε*(*λ*) (measured in bits) from the true unknown Poisson distribution to the A-C statistic (solid line) and its analytical approximation (20) (dashed line).

Figure 2 presents values of the expected divergence *ε*(*λ*) (measured in bits) calculated numerically from the definition (9), as well as their analytical approximation calculated from (20). As expected, the two curves are in good correspondence, as our approximation is *O*(*λ*^-3^).

Results of this section suggest that if the true Poisson source *P*(·|*λ*) is not known, the A-C statistic (*y|x*), *based on a single observed tag count realization x *from *P*(·|*λ*), is on average not further away from the truth *P*(*y|λ*) than half a bit of additional information. As the mean tag count *λ *increases, so does the uncertainty in the generating Poisson distribution *P*(·|*λ*). As a consequence, the average K-L divergence *ε*(*λ*) from *P*(·|*λ*) to the approximating A-C statistic (based on a single realization from *P*(·|*λ*)) gets larger. The average K-L divergence expressed in bits increases with increasing *λ *from about 0.42 bits to 0.5 bits.

## Results and Discussion

The Audic-Claverie method [1] has been and still continues to be a popular approach for detection of differentially expressed genes in the SAGE framework. The method is based on the assumption that under the null hypothesis the tag counts *x, y *in two libraries come from the same but unknown Poisson distribution *P*(·|*λ*). The problem is that each SAGE library represents only a single measurement. We have rigorously analyzed usefulness of the Audic-Claverie method by investigating the A-C statistic (*y|x*) that forms a backbone of the method and represents our knowledge of the underlying Poisson distribution *P*(·|*λ*) based on only one tag count *x *drawn from it.

It turns out that the Poisson distribution is rather "rigid" in the sense that it is unimodal and parametrized by a single parameter *λ *representing both its mean and variance. Learning about *P*(·|*λ*) from a very limited sample (as one is effectively bound to do in the SAGE framework) is much less suspicious than one might naively expect.

We have first shown that the A-C statistic (*y|x*), even though not a Poisson distribution itself, naturally captures the distribution of further tag counts *y*, given a single observation *x *from the unknown *P*(·|*λ*). According to Theorem 1, for integer λ, both (·|*x*) and *P*(·|*λ*) have two neighboring modes with decreasing probability values as one moves away from the modes in either direction. In particular, *P*(·|*λ*) has the modes located at *λ *and *λ *- 1, with *P*(*λ*|*λ*) = *P*(*λ *- 1|*λ*). Given a tag count *x *≥ 1, (*y|x*) has the modes located at *x *and *x *- 1, with (*x|x*) = (*x *- 1|*x*).

We then analyzed how 'close' is the A-C statistic (·|*λ*) (in terms of K-L divergence) to the underlying Poisson distribution *P*(·|*λ*) of tag counts. It turns out that the K-L divergence from *P*(*y|λ*) to (*y|x*) is minimized at the mode of *P*(*y|λ*), i.e. when *x *= *λ *- 1 (Theorem 2). Most importantly, by Theorem 4, on average, the A-C statistic is never too far from the true underlying distribution. To be precise, up to terms of order *O*(*λ*^-3^), on average, the A-C statistic is never further away from the truth *P*(·|*λ*) than half-a-bit of additional information. Hence, the Audic-Claverie method can be expected to work well even though the SAGE libraries represent very sparse samples.

So far the Audic-Claverie methodology for detection of differentially expressed genes has been verified only empirically through a series of specific Monte Carlo simulations [1]. It has not been clear how general the apparently stable simulation findings were. Besides detailed explanations of the nature of A-C statistic capturing the unknown Poisson distribution based on single observation only, we showed that the A-C statistic is *universally *applicable in any situation where inferences about the underlying Poisson distribution must be made based on an extremely sparse sample. Such situations are referred to in machine learning as 'one-shot-learning'. In the Monte Carlo simulations of [1] the false alarm rate was small for genes associated with small tag counts and gradually increased for higher tag counts. The false alarm rate, however, never exceeded the significance level of the test. These findings are consistent with the theoretically calculated divergence function *ε*(*λ*) (eq. (20)) illustrated in figure 2. With increasing mean tag count λ, it is more likely that increased counts *x *will be observed. But as *λ *increases, so does the uncertainty in the generating Poisson distribution *P*(·|*λ*). Consequently, the average K-L divergence *ε*(*λ*) from *P*(·|*λ*) to the approximating A-C statistic (based on a single realization *x *from *P*(·|*λ*)) gets larger. For smaller *λ *the underlying Poisson distribution is well captured by the A-C statistic and the test that operates on it will be well behaved. As *λ *grows, the average K-L divergence *ε*(*λ*) saturates at 0.5 bits implying that the test based on the A-C statistic will continue to be well behaved even for large values of the mean tag count *λ*.

The Audic-Claverie method has also been formulated for the case of two cDNA libraries of unequal size. Similar methodologies have been proposed for the case of multiple cDNA libraries (e.g. [7]). Even though developed under the limited assumption of two libraries of the same size, theoretical results obtained in this paper offer deep insights into the workings of the Audic-Claverie approach and provide an information theoretic justification for its use when analyzing expression patterns in cDNA arrays. Of course, when using libraries of unequal size, the A-C statistic will no longer be symmetric, putting more weight on the more populated library. Information theoretic investigation of statistics developed for pattern analysis in the cases of unequal multiple libraries is a matter for our future work.

## Conclusion

Detection of differentially expressed genes is a crucial step in any large scale automated analysis of patterns of gene expression data. One of the most popular techniques for identifying genes with statistically different expression in SAGE libraries is the methodology of Audic and Claverie [1]. The methodology relies on learning the underlying Poisson distribution of tag counts from a single observation from it in the form of (A-C statistic). In this paper we rigorously analyzed the A-C statistic. We have shown that under the null hypothesis of not differentially expressed genes:

1. The A-C statistic and the underlying Poisson distribution share the same mode structure.

2. The K-L divergence from the true unknown Poisson distribution to the A-C statistic is minimized when the A-C statistic is conditioned on the mode (not mean) of the Poisson distribution.

3. The expected K-L divergence from the true unknown Poisson distribution to the A-C statistic is never larger than 1/2 bit, irrespective of the mean of the Poisson distribution.

4. The expected K-L divergence from the true unknown Poisson distribution to the A-C statistic can be approximated up to order *O*(*λ*^-3^) by a simple function of the form *a*_0 _+ *a*_1_*λ*^-1 ^+ *a*_2_*λ*^-2^. For the divergence measured in bits, *a*_0 _= 1/2, *a*_1 _= 1/24 and *a*_2 _= 1/32.

Even though the A-C statistic infers the unknown underlying Poisson distribution based on one count observation only, the Audic-Claverie method should work reasonably well in most cases, since under the null hypothesis, the average divergence from the unknown Poisson distribution to the A-C statistic is guaranteed not to exceed 1/2 bit. This constitutes a rigorous quantitative argument, extending the empirical Monte Carlo studies of [1], that supports the wide spread use of Audic-Claverie method, even though by their very nature, the SAGE libraries represent very sparse samples.

## Authors' contributions

I am the sole author of this paper.
